# The significance of ^18^F-FDG PET/CT in secondary hemophagocytic lymphohistiocytosis

**DOI:** 10.1186/1756-8722-5-40

**Published:** 2012-07-23

**Authors:** Li-Juan Zhang, Ji Xu, Peng Liu, Chong-Yang Ding, Jian-Yong Li, Hong-Xia Qiu, Su-Jiang Zhang

**Affiliations:** 1 Department of Hematology, The First Affiliated Hospital of Nanjing Medical University, Jiangsu Province Hospital, 300 Guangzhou Road, Nanjing, China; 2 Department of Hematology, Huai’an First People’s Hospital, Nanjing Medical University, Huai’an, China; 3 Department of PET/CT Center, The First Affiliated Hospital of Nanjing Medical University, Jiangsu Province Hospital, Nanjing, China

## Abstract

This study was aimed to investigate the significance of ^18^F-FDG PET/CT in secondary hemophagocytic lymphohistiocytosis (sHLH) patients. A total of 18 patients received ^18^F-FDG PET/CT scan at initial diagnosis. All patients (18/18) had at least 3 organs involved, with increased FDG metabolism in different degrees. Fifteen cases (15/18) had definite underlying diseases, including infections (IAHLH), rheumatosis (RAHLH), or malignancy (MAHLH). The SUV_max_ of patients in MAHLH group was significantly higher than patients in IAHLH group or RAHLH group (P = 0.015, P = 0.045). Furthermore, the SUV_max_ of patients in IAHLH group was significantly higher than patients of RAHLH group (P = 0.043). Therefore, we concluded that ^18^F-FDG PET/CT may especially play important role in differential diagnosis of sHLH.

## To the Editor

Secondary hemophagocytic lymphohistiocytosis (sHLH) is a hyper-inflammatory clinical syndrome mainly caused by severe infections, autoimmune inflammatory disorders and malignancies, especially lymphoma [1-3]. Up to date, very few data from the literature are available regarding the role of ^18^F-FDG PET/CT in sHLH. In this study, 18 of 50 patients with sHLH who were admitted into our hospital between May 2007 and December 2010 underwent the examination (Table 1). The male-to-female ratio was 1:1, and the median age was 35 years (15-73). The diagnosis of HLH was made according to HLH-2004 diagnostic guidelines [4,5], and the underlying diseases were confirmed by a series of pathogenesis examinations including pathology, immunology, bacterial culture and virus detection et al. The maximum standardized uptake values (SUV_max_) used to measure the level of FDG uptake were determined in all lesions [6]. All of the 18 patients had at least 3 organs involved, with increased FDG uptake at different level, including 18 cases showing splenomegaly, 16 cases serous effusions, 16 cases lymphadenopathy, 13 cases bone lesions, 12 cases pneumonia, 8 cases hepatomegaly, 5 cases brain parenchymal or cerebroventricular lesions, 5 cases cholecystitis, 4 cases myocardium lesions, and 2 cases kidney calculi. There were also other organs involved, such as larynx, muscles and adnexauteri. Fifteen patients (15/18) had definite underlying diseases, and were divided into three groups.,including Infection Associated HLH (IAHLH, including EBV-HLH, n = 8), Rheumatosis Associated HLH (RAHLH, n = 2), and Malignancy Associated HLH (MAHLH, n = 5). The SUV_max_ of patients in MAHLH group was significantly higher than those of patients with IAHLH (Mean 12.0 *vs.* 6.8, *P* = 0.015), and RAHLH (Mean 12.0 *vs.* 2.7, *P* = 0.045). Furthermore, the SUV_max_ of patients with IAHLH was significantly higher than that of patients with RAHLH (Mean 6.8 *vs.* 2.7, *P* = 0.043). However, no significant difference in survival time was found between the three different sHLH subtype according to Kaplan-Meier analysis (*P* >0.05). In conclusion, ^18^F-FDG PET/CT may play important role in differential diagnosis of sHLH, with high SUV pointing toward underlying malignancy.

**Table 1:** Characteristics of 18 sHLH patients

**No.**	**Age/ Sex**	**Underlying disease**	**Therapy**	**Outcome**	**Survival (month)**	**Organs**	**SUV**_**max**_
1	35/M	Lymphoma (NK / T)	IVIG/HLH-2004 regimen(1 cycle) → High-dose methylprednisolone pulse therapy	Died of intracranial hemorrhage	1.7	6	12.3
2	35/F	Lymphoma (NK / T)	The Hyper-CVAD regimen (1 cycle)	Died of intracranial hemorrhage	1.2	6	15.7
3	18/M	Lymphoma (NK / T)	The CHOP regimen(1 cycle)	Died of acute hemorrhage of gastrointestinal tract	0.3	7	14.6
4	56/M	Lymphoma	Hydrocortisone 100mg×5d	Died of intracranial hemorrhage	1.7	5	4.3
5	32/M	Lymphoma	Dex 10mg/d×3d	Died of liver failure	0.3	10	13.3
6	37/F	Sjögren's syndrome	The COP regimen(3 cycle)	CR	>12	5	0.7
7	15/F	UCTD	The COP regimen (4 cycle)	CR	>45	3	4.6
8	21/F	EBV infection	HLH-2004 regimen (1 cycle)	Died of acute hemorrhage of gastrointestinal tract	1.7	7	6.6
9	17/M	EBV infection	Methylprednisolone 40 mg/d×24d	CR	>22	7	8.3
10	46/M	EBV infection	Dex 15mg/d×4d	Died of septic shock	0.4	6	10
11	73/M	EBV infection	The COP regimen (7 cycle)	Died of multi-organ failure	6	7	5.2
12	26/F	CMV infection	IVIG/HLH-2004 regimen (1 cycle) →The CHOP regimen(2 cycle)	CR	>24	6	9
13	24/F	CMV infection	The COP regimen (7 cycle)	Died of respiratory failure	2.2	5	4.2
14	69/F	MRSH infection	The COP regimen (2 cycle)	Died of respiratory failure	2.0	6	5.2
15	62/F	Fungal Infection	The COP regimen (7 cycle)	stable	>8	4	5.8
16	44/F	Malignant tumour?	Methylprednisolone 40 mg/d×5d	Died of multi-organ failure	0.4	8	7.7
17	56/M	Lymphoma?	The CHOP regimen (2 cycle) →Splenectomy→The Hyper-CVAD regimen (1cycle)	stable	>13	6	5.7
18	18/M	indefinite	HLH-2004 regimen (1 cycle)	Died of intracranial hemorrhage	0.2	3	4.2

HLH-2004, dexamethasone, etopside and Ciclosporin A; CHOP, cyclophosphamide, adviamycin, vincristine and prednisolone; COP, cyclophosphamide, vincristine and prednisone; Hyper-CVAD, cyclophosphamide, vincristine, doxorubicin, dexamethasone, methotrexate and cytarabine; DEX, dexamethasone; CR, complete response; UCTD, undifferentiated connective tissue disease; EBV, Epstein-Barr virus; CMV, cytomegalovirus; MRSH, methicillin-resistant Staphylococcus hominis.
